# High SOX8 expression promotes tumor growth and predicts poor prognosis through GOLPH3 signaling in tongue squamous cell carcinoma

**DOI:** 10.1002/cam4.3041

**Published:** 2020-04-19

**Authors:** Shuwei Chen, Huan Li, Xiyuan Li, Wenkuan Chen, Xing Zhang, Zhongyuan Yang, Zhipeng Chen, Jingtao Chen, Ying Zhang, Dingbo Shi, Ming Song

**Affiliations:** ^1^ Department of Head and Neck Surgery Sun Yat‐sen University Cancer Center Guangzhou China; ^2^ State Key Laboratory of Oncology in South China Guangzhou China; ^3^ Collaborative Innovation Center for Cancer Medicine Guangzhou China; ^4^ Department of Intensive Care Unit Sun Yat‐sen University Cancer Center Guangzhou China; ^5^ Department of Experimental Research Sun Yat‐sen University Cancer Center Guangzhou China

**Keywords:** GOLPH3, SOX8, tongue squamous cell carcinoma, tumor growth

## Abstract

According to our previous study, GOLPH3 is markedly up‐expressed in tongue squamous cell carcinoma (TSCC), which is also associated with poor survival. However, it remains unclear about key upstream and downstream mechanisms of GOLPH3. This study aimed to illuminate new mechanisms modulating GOLPH3 upregulation and promoting TSCC development at the molecular level. Using mass spectrometry and agarose‐streptavidin‐biotin pull‐down analyses, SOX8 (SRY‐Box 8) was identified to be the new protein to bind the GOLPH3 promoter within TSCC cells, which was further verified to be the regulator of GOLPH3 upregulation. The knockdown of SOX8 suppressed the promoter activity of GOLPH3, while secondarily inhibiting TSCC cell proliferation both in vivo and in vitro. Interestingly, GOLPH3 overexpression rescued the SOX8 knockdown‐mediated suppression on TSCC proliferation. Additionally, exogenous over‐expression of SOX8 also activated the activity of promoter as well as GOLPH3 expression, in the meantime of promoting TSCC development. Moreover it was discovered that SOX8 regulated GOLPH3 expression through interacting with TFAP2A. Moreover our results suggested that the SOX8 level was increased within tumor tissue compared with that in para‐cancer normal counterpart, which showed positive correlation with the GOLPH3 level. According to Kaplan‐Meier analyses, TSCC cases having higher SOX8 and GOLPH3 expression were associated with poorer prognostic outcomes. Taken together, this study reveals that SOX8 enhances the TSCC cell growth via the direct transcriptional activation of GOLPH3, which also indicates the potential to use SOX8/GOLPH3 pathway as the treatment target among TSCC patients.

## Graphical abstract

SOX8 promotes TSCC cell growth and predicts poor prognosis via the direct transcriptional activation of GOLPH3 expression, which suggests that the SOX8/GOLPH3 pathway may be a potential therapeutic target for TSCC patients.

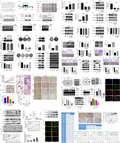

## 1 INTRODUCTION

Oral carcinoma has been recognized as one of the most common malignancies worldwide. Some population studies suggested that, tongue is most susceptible to cancer in the intraoral site, whereas squamous cell carcinoma is the most frequently seen oral carcinoma pathological type.^1^ Tongue squamous cell carcinoma (TSCC) is frequently associated with the complications of serious speech defect, deglutition, mastication, and cancer‐related deaths.^2^ Although we have made significant progresses in treatment and prevention, the mortality and morbidity of TSCC are still high, which even show an increasing trend in some developing countries. Over the past 30 years, the long‐time survival of tongue carcinoma patients, especially for the advanced cases, has not been substantially improved, with tongue being one of the frequently occurring site of cancer.^3^ Therefore, it is of great significance to further explore the molecular mechanisms underlying the tumorigenesis and progression of TSCC and to identify the novel therapeutic targets for TSCC.

As one of the greatly conservative 34‐kDa protein, Golgi phosphoprotein 3 (GOLPH3) is initially identified in the Golgi apparatus.^4^ Its encoding gene is located on human chromosome 5p13.^5^ GOLPH3 up‐expression is first detected in human lung cancer specimens, which is triggered within malignancies possessing 5p amplification. Besides, GOLPH3 is further confirmed in study as the oncogene that has powerful transformation activity.^6^, ^7^ GOLPH3 is found to act as a key factor in mTOR activation in the non‐small cell lung carcinoma (NSCLC), which is ascribed to the correlation of 5p amplification with the increased phosphorylation of mTOR substrate.^8^ Such result indicates that GOLPH3 induces cell transformation and carcinogenesis through activating the mTOR complex (mTORC).

Multiple studies confirm that GOLPH3 participates in vesicular trafficking and glycosylation, which are the potential carcinogenic mechanisms. Recently, GOLPH3 is recognized to be the oncogene within ovary carcinoma,^9^ colorectal carcinoma,^10^ breast cancer,^11^ melanoma,^12^ prostate cancer,^13^ rhabdomyosarcoma,^14^ and glioma.^15^ Over‐expression of GOLPH3 is believed to enhance the proliferation and growth of cancer cells, which is achieved by activating the nuclear factor kappa B (NF‐B), Wnt, and mTOR signal transduction pathways.^16^ Nonetheless, it remains largely unclear about the mechanism of the elevated GOLPH3 expression in cancer cells. Firstly, the GOLPH3 promoter region is detected, whose transcriptional factors or regulatory factors regulate tumor development and progression.

Proteins in the sex‐deciding region on the Y chromosome (SRY)‐associated high mobility group box (SOX) family are demonstrated to participate within reprogramming, regeneration^17^, ^18^ and other pathological processes.^19^ Thus far, the SOX family proteins include 20 different subtypes termed from A to H, and they share the HMG domain that has high sequence identity (over 80%). SOX8, SOX9 and SOX10 are the E subgroup members in the SOX family genes, among which, the latter two subtypes have been confirmed to be implicated in specific human disorders.^20^, ^21^ It is observed in one study that, SOX8 participates in the development of mammalian testis.^22^ Recently, studies discover that SOX8 is overexpressed in brain tumor^23^ and hepatocellular carcinoma (HCC).^24^ Nonetheless, the expression level and function of SOX8 in TSCC are still unknown. The structure and genomic organization of SOX8 are analogous to those of SOX9, a gene that is also over‐expressed in numerous cancers.^25^, ^26^, ^27^, ^28^, ^29^ As a result, we hypothesize thatSOX8mayparticipate in other biochemical process other than regulating the gene expression at post‐transcriptional level. Here, the biotin‐streptavidin‐agarose pull‐down assay was carried out to analyze several common binding proteins on the promoter DNA sequence in GOLPH3 promoter region within TSCC cells. SOX8 was confirmed to be the new protein binding to the GOLPH3 promoter region by chromatin immunoprecipitation (ChIP) assay; besides, SOX8 was validated to bind to endogenous promoter region of GOLPH3 within the TSCC cells. The new molecular role of SOX8 had been ascertained, which bound to GOLPH3 promoter, and regulated GOLPH3 expression and cell proliferation. Therefore, our study demonstrated the potential of using SOX8/GOLPH3 signal transduction pathway to be the new target in TSCC therapy.

## 2 RESULTS

### 2.1 SOX8 had been recognized to be the protein binding to GOLPH3 promoter within the TSCC cell lines

**FIGURE 1:**
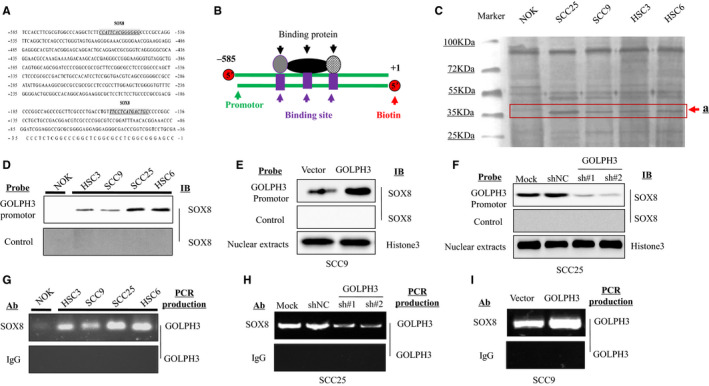
SOX8 is recognized to be a protein binding to GOLPH3 promote within the tongue squamous cell carcinoma (TSCC) cell lines. Numerous transcriptional factor‐binding sites of SOX8 are identified in the core promotor region of GOLPH3 (A). The 5'‐biotin conjugated DNA probe (585‐bp) corresponding to GOLPH3 promoter region comprising the nucleotides −585 to +1 is synthesized (B). The GOLPH3 promoter‐binding proteins band levels (about 30 kDa) are markedly increased within the TSCC cell lines (SCC25, SCC9, HSC3, and HSC6), compared with the normal oral epithelium (NOK) cells (C). More SOX8 is bound onto GOLPH3 promoter probe within the TSCC cell lines (SCC25, SCC9, HSC3, and HSC6), compared with NOK cells (D). ChIP assay finds that SOX8 strongly binds onto GOLPH3 promoter region among the four TSCC cell types (SCC25, SCC9, HSC3, and HSC6), in comparison with NOK cells (E). SOX8 binding onto GOLPH3 promoter probe is elevated within SCC9 cells with SOX8 over‐expression (F), nonetheless, it is reduced within SCC25 cells with SOX8 knockdown (G). ChIP assay confirms that SOX8 is weakly bound onto GOLPH3 promoter within SCC25 cells with SOX8 knockdown (H), but it is strongly bound onto GOLPH3 promoter within SCC9 cells with SOX8 over‐expression (I)

At first, the schematic diagram of GOLPH3 promoter comprising the nucleotides −585 to +1 was analyzed, which predicted the transcriptional factors binding sites of SOX8 (Figure 1A). Afterwards, the structures of the 5’‐biotin‐conjugated GOLPH3 promoter probe (−585 to +1 bp), together with its related promoter‐binding proteins, were simulated (Figure 1B).

Afterwards, this biotin‐labeled promotor probe was incubated with the nuclear proteins collected based on4 kinds of human TSCC cells (namely, SCC25, SCC9, HSC3, and HSC6) as well as the normal oral epithelium cells (NOK), so as to knock out proteins binding to the GOLPH3 promoter, followed by isolation through SDS‐PAGE. Then, the protein gel was stained with silver, which revealed markedly increased protein band levels (about 35 kDa) within the TSCC cell lines (SCC25, SCC9, HSC3 and HSC6), compared with that in NOK cells (Figure 1C, arrow a).

Besides, western blotting was carried out to examine the SOX8 protein expression within TSCC cell lines. In addition, the anti‐SOX8 antibody was also used in Western Blotting to detect SOX8 binding onto the non‐specific probe (NSP) or 5'‐biotin conjugated GOLPH3 promoter probe. The SOX8 protein expression had also been determined using GOLPH3 probe streptavidin bead‐nuclear protein complexes within SCC9, HSC3, SCC25 as well as HSC6 cell lines; however, SOX8 was rarely expressed within the NOK cell line (Figure 1D). Furthermore, western blotting was also conducted in SCC9 cells transfected with GOLPH3 over‐expression plasmids, so as to detectSOX8 binding onto the NSP or 5'‐biotin conjugated GOLPH3 promoter probe. SOX8 protein expression had been higher in cells with GOLPH3 over‐expression (Figure 1E). Also, SOX8 binding onto NSP or 5'‐biotin conjugated GOLPH3 promoter probe was also detected in SCC25 cells transfected with GOLPH3 shRNA plasmids and scratch shRNA. Our results suggested that, SOX8 protein expression was down‐regulated in cells transfected with GOLPH3 shRNA plasmids (Figure 1F). Additionally, our ChIP results also showed that SOX8 was more strongly bound to the GOLPH3 promoter in the SCC9, HSC3, SCC25, and HSC6 cell lines; by contrast, SOX8 was rarely expressed within the NOK cell line (Figure 1G). Consistently, it was also found that SOX8 showed weak binding onto GOLPH3 promoter within the GOLPH3‐knockdown SCC25 cell line (Figure 1H), whereas strong binding onto GOLPH3 promoter within SCC9 cells with GOLPH3over‐expression (Figure 1I).

### 2.2 SOX8 increased GOLPH3 expression and the promoter activity within the TSCC cell lines

**FIGURE 2:**
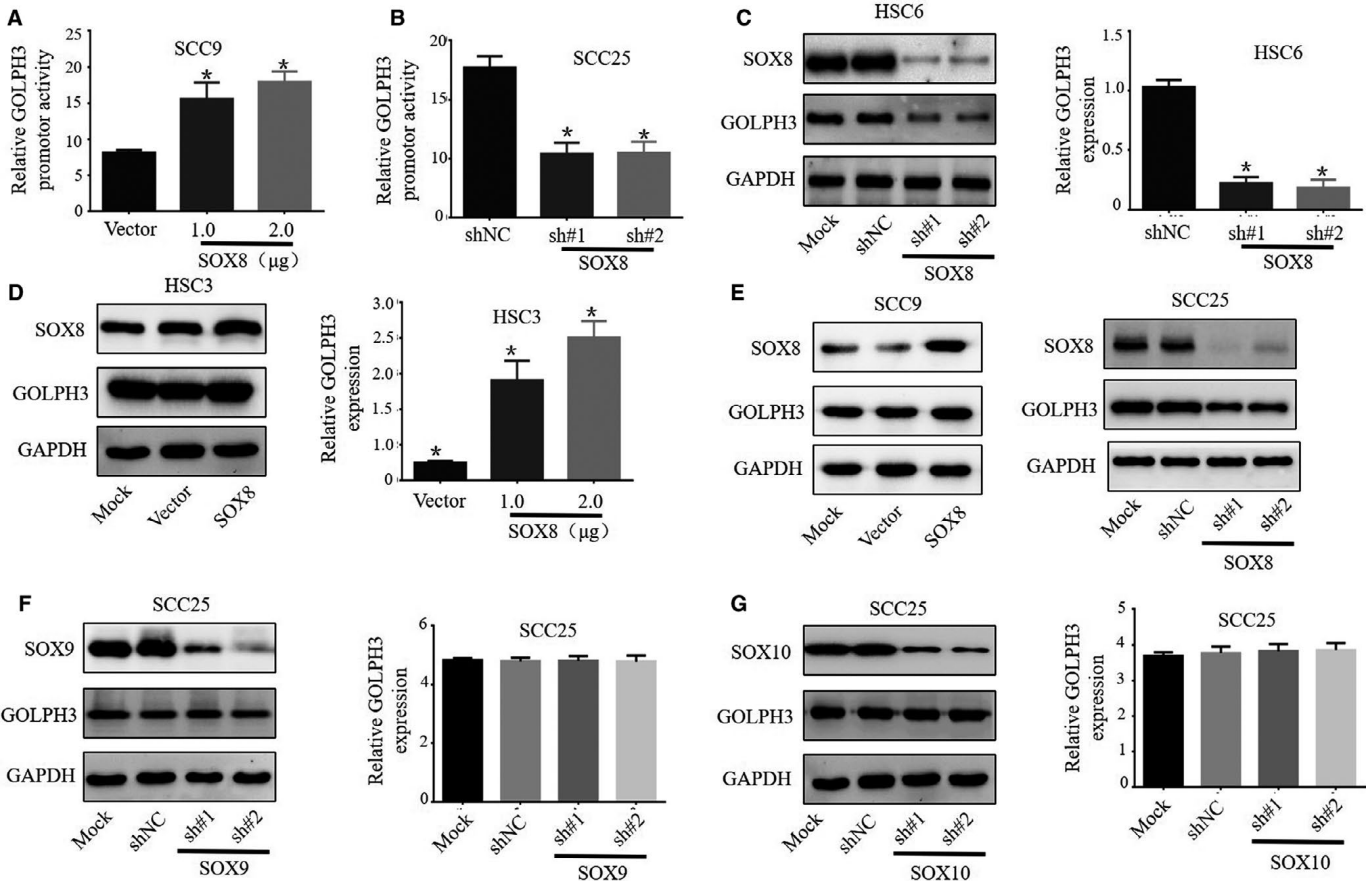
SOX8 increased GOLPH3 expression and promoter activity within the tongue squamous cell carcinoma (TSCC) cell lines. Luciferase reporter gene assay finds that, SOX8 over‐expression enhances activity of GOLPH3 promoter within SCC9 and HS3 cells, compared to the vector‐treated group (A), whereas SOX8 knockdown dramatically decreases activity of GOLPH3 promoter within SCC25 as well as HSC6 cells, compared with groups treated with nonspecific shRNA (shNC) (B). SOX8 knockdown is found to inhibit GOLPH3 expression at the translational and transcriptional levels within HSC6 (C), while SOX8 over‐expression enhances the GOLPH3 mRNA and protein levels in HSC3 cells (D). Also, western blotting is carried out, which confirms that SOX8 knockdown inhibits GOLPH3 expression in SCC25 cells, and that SOX8 over‐expression enhances the activity of GOLPH3 promoter in SCC9 cells (E). Besides, western blotting and RT‐qPCR show that neither SOX9 (F) nor SOX10 (G) regulates GOLPH3 expression in TSCC cells at transcriptional and translational levels

The plasmids over‐expressing SOX8 were established, and 2 SOX8 short‐hairpin RNAs (sh#1 and sh#2) were also synthesized. The luciferase reporter gene assay was performed, which revealed that SOX8 over‐expression evidently increased the activity of GOLPH3 promoter within SCC9 cells, compared with that of vector‐treated counterpart (Figure 2A), whereas SOX8 knockdown dramatically reduced the activity of GOLPH3 promoter within SCC25 cells, compared with that in cells treated with nonspecific shRNA (shNC) (Figure 2B). It was found that SOX8 knockdown inhibited GOLPH3 level at both translational and transcriptional levels within HSC6 cells (Figure 2C), whileSOX8 over‐expression increased the GOLPH3 mRNA and protein levels within HSC3 cells (Figure 2D). Further, Western Blotting was conducted, which confirmed that SOX8 knockdown inhibited GOLPH3 expression in SCC25 cells, whereas SOX8 over‐expression enhanced GOLPH3 expression in SCC9 cells (Figure 2E). In addition, the effect of SOX9 or SOX10 (the SOX8 family proteins) on regulating GOLPH3 expression was also analyzed. The results showed that neither SOX9 (Figure 2F) nor SOX10 (Figure 2G) regulated GOLPH3 expression in TSCC cells.

### 2.3 SOX8 promoted TSCC development through GOLPH3 signal transduction pathway in vitro

**FIGURE 3:**
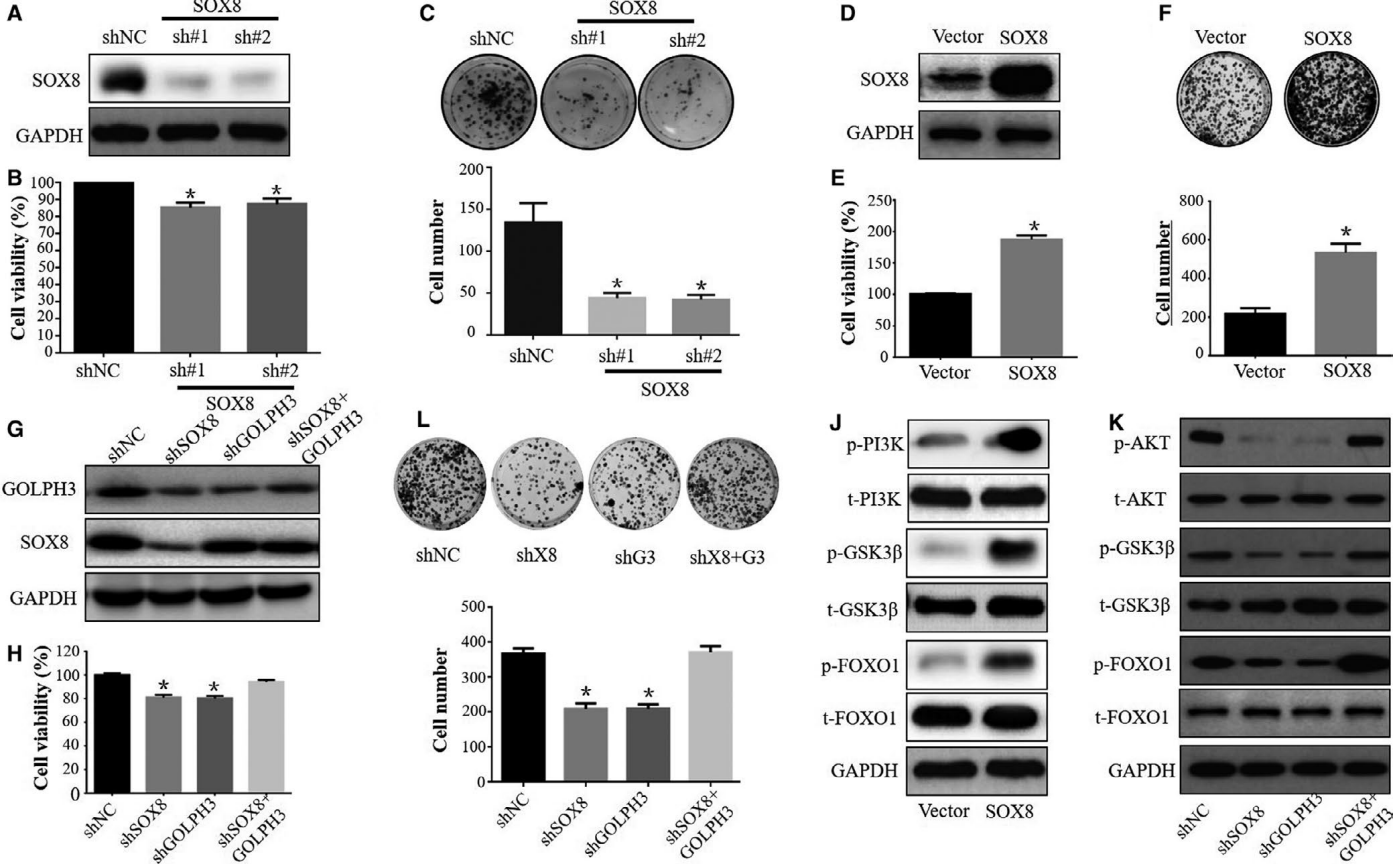
SOX8 promotes tongue squamous cell carcinoma (TSCC) growth via the GOLPH3 signaling pathway in vitro*.* Western blotting confirms SOX8 knockdown in SCC25 cells by SOX8‐specific shRNAs (sh#1 and sh#2) (A). SOX8 knockdown decreases the viability (B) and colony‐forming capacity of SCC25 cells (C). Western Blotting confirms the over‐expression of SOX8 in SCC25 cells (D). SOX8 over‐expression promotes the proliferation and viability (E), and the colony‐forming capacity (F) of SCC25 cells. In SOX8‐depleted cells, GOLPH3 over‐expression rescues the GOLPH3 protein expression (G), together with cell viability (H) and colony forming capacity (I). Moreover western blotting suggests that SOX8 over‐expression up‐regulates the activation of p‐PI3K, p‐GSK3β, and p‐FOXO1, but not the total expression of PI3K, GSK3β, and FOXO1 in SCC9 cells (J). Immunoblotting test indicates that GOLPH3 over‐expression rescues the protein expression of p‐AKT, p‐GSK3β, and p‐FOXO1, which is markedly down‐regulated following SOX8 knockdown, respectively, in SCC25 cells (K)

Cell lines with stable SOX8 over‐expression (SCC9) and stable SOX8 knockdown (SCC25) were constructed. Our results suggested that, the knockdown of SOX8 expression in SCC25 cells by SOX8‐specific shRNAs(sh#1 and sh#2) (Figure 3A) markedly decreased the colony forming capacity (Figure 3C) and viability (Figure 3B) of TSCC cells. Also, it was discovered that, the over‐expression of SOX8 (Figure 3D) effectively promoted the proliferation and viability (Figure 3E), as well as the colony forming capacity (Figure 3F) of SCC25 cells. Moreover the GOLPH3 level restoration assays were then carried out to over‐expressGOLPH3 within the SOX8‐free SCC25cells. According to our results, GOLPH3 expression had been downregulated within SOX8‐free cells (Figure 3G, lane2) or in the GOLPH3‐knockdown cells (Figure 3G, lane3), while GOLPH3 over‐expression rescued GOLPH3 expression in the SOX8‐depleted cells (Figure 3G, lane4). The above findings demonstrated that, either exogenous or endogenous SOX8 expression regulated GOLPH3 expression. Results of cell proliferation and viability assay (Figure 3H), together with colony forming assay (Figure 3I) revealed that theSOX8‐mediated inhibition of cell proliferation was reversed by GOLPH3 over‐expression in the SOX8‐depleted SCC25 cells (Figure 3H and I).

Furthermore, SOX8 effect on key proteins within theGSK3β/FOXO1 and PI3K/Akt signal pathway, the critical GOLPH3 signaling‐associated downstream pathway that affected cell proliferation,^11^ was assessed. Our results found that SOX8 over‐expression up‐regulated the activation of p‐PI3K, p‐GSK3β, andp‐FOXO1, but not the total expression of PI3K, GSK3β, and FOXO1 in SCC9 cells (Figure 3J). Finally, GOLPH3 level restoration assays were carried out within SOX8‐free SCC25 cells. These pivotal proteins were detected by immunoblotting test, and GOLPH3 over‐expression rescued the expression of p‐AKT, p‐GSK3β, and p‐FOXO1 proteins in SCC25 cells (Figure 3K), which was markedly down‐regulated following SOX8 or GOLPH3 knockdown, respectively (Figure 3K).

### 2.4 SOX8 regulated the invasion and migration of TSCC cells via GOLPH3

**FIGURE 4:**
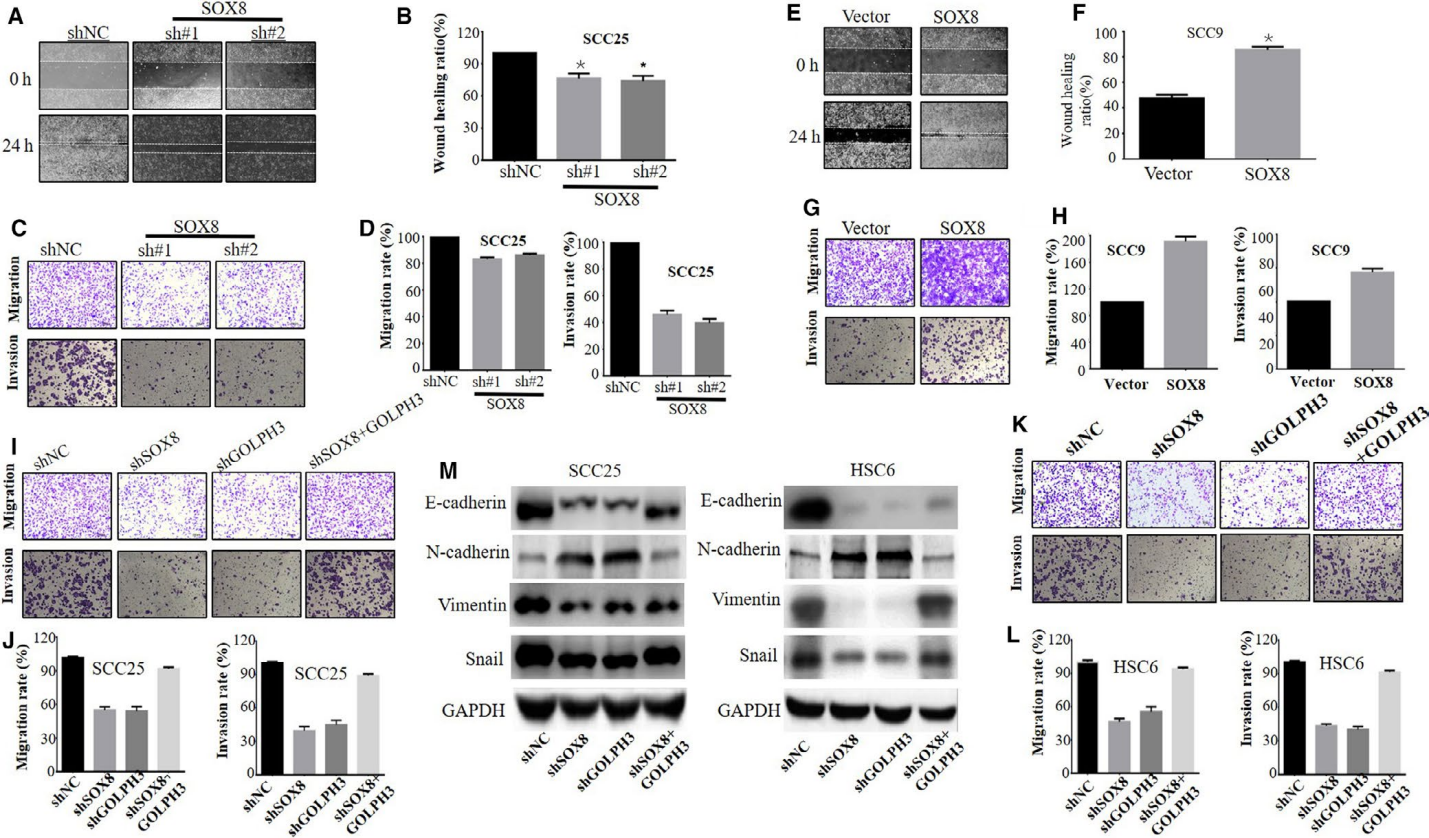
SOX8 regulates the invasion and migration of tongue squamous cell carcinoma (TSCC) cells via GOLPH3. SOX8 knockdown remarkably inhibits the wound healing rate (A and B), as well as migration and invasion rates (C and D) in SCC25 cells. Inversely, SOX8 over‐expression increases the wound healing rate (E and F), together with the migration and invasion rates (G and H) of SCC9 cells. It is also found that GOLPH3 knockdown also evidently inhibited the invasion and migration of SCC25 (I and J) and HSC6 cells (K and L). But, GOLPH3over‐expression rescues the migration and invasion rates in SOX8‐depleted cells. Western blotting finds that, only SOX8 knockdown or GOLPH3 knockdown notably down‐regulates the protein expression of β‐catenin, E‐cadherin, Vimentin, Snail, and c‐Myc in SCC25 and HSC6 cells. However, the over‐expression of GOLPH3 in cells with stable SOX8 knockdown distinctly antagonized β‐catenin, Vimentin, E‐cadherin, c‐Myc, and Snail protein expression (M)

SOX8 functions during TSCC cell wound healing, invasion and migration were investigated through the Transwell and wound healing assays. As suggested by our results, SOX8 knockdown remarkably suppressed the rate of wound healing in SCC25 cells (Figure 4A and B). Besides, the Transwell assay results showed that SOX8 knockdown inhibited the SCC25 cell invasion and migration rates (Figure 4C and D). Inversely, SOX8 over‐expression markedly increased the wound healing rate in SCC9 cells, compared with that in vector plasmid‐treated group (Figure 4E and F). Furthermore, SOX8 over‐expression was also discover to enhance SCC9 cell invasion and migration (Figure 4G and H).

Moreover SOX8 was confirmed to regulate the wound healing, invasion and migration capacities in TSCC cells via GOLPH3 activation. Our data showed that the over‐expression of GOLPH3 in SCC25 cells with stable SOX8 knockdown boosted the invasion and migration capacities of cells in comparison with those in SOX8‐knockdown cells under control vector treatment (Figure 4I and J). Moreover the over‐expression of GOLPH3 in HSC6 cells with stable SOX8 knockdown distinctly reversed the inhibition of SOX8 knockdown on cell invasion and migration (Figure 4K and L). Additionally, GOLPH3 knockdown within the SCC25 and HSC6 cell lines markedly suppressed cell migration and invasion, compared with the shNC‐treated group (Figure 4I and K).

Epithelial‐mesenchymal transition (EMT) plays an important role in tumor genesis and metastasis. Therefore, it was speculated thatSOX8might be involved in EMT through the activation of GOLPH3 signaling. The EMT signaling‐related proteins, including E‐cadherin, N‐cadherin, Vimentin, and Snail, were examined for their expression to solve the above question. According to our findings, SOX8 and GOLPH3 knockdown, which were achieved using specific shRNAs, notably down‐regulated the protein levels of E‐cadherin, Vimentin, and Snail in SCC25 and HSC6 cells, relative to the respective control groups (Figure 4M). In addition, over‐expression of GOLPH3 in cells with stable SOX8 knockdown distinctly antagonized the down‐regulated protein levels of E‐cadherin, Vimentin, Snail, and c‐Myc induced by SOX knockdown (Figure 4M).The above results demonstrated that, GOLPH3 exerted the functional part during SOX8‐induced EMT process and in TSCC cell invasion and migration.

### 2.5 SOX8 boosted TSCC tumor metastasis and development via GOLPH3

**FIGURE 5:**
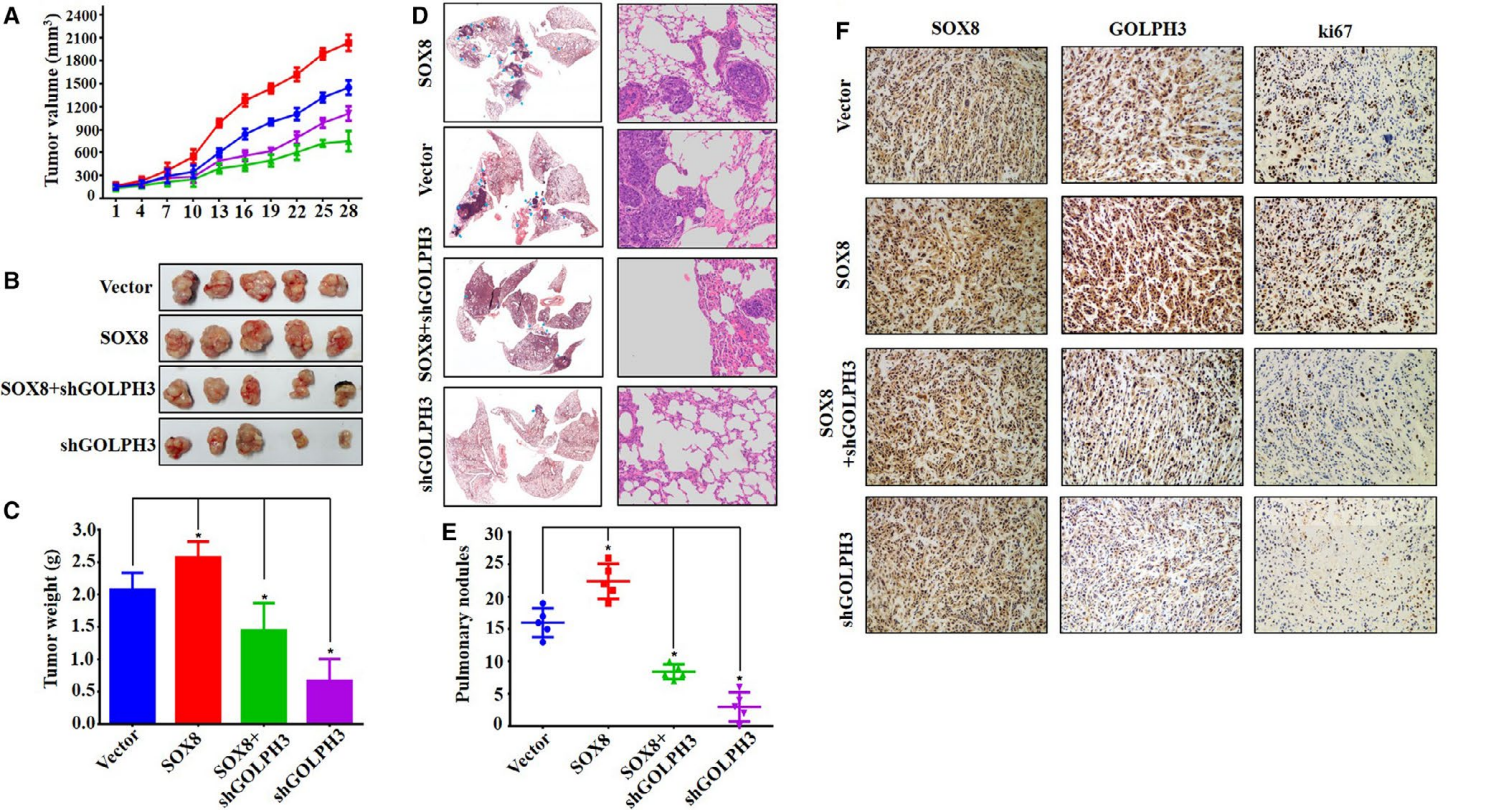
SOX8 promotes tongue squamous cell carcinoma (TSCC) tumor growth and metastasis via GOLPH3. SCC25 xenografts with SOX8 over‐expression grow fastest than those in other groups. In contrast, SCC25 xenografts with GOLPH3 knockdown grow at the slowest rate (A), but SCC25 xenografts with SOX8 over‐expression combined with GOLPH3 knockdown grow lower than those with SOX8 over‐expression alone. The ranks of tumor size and weight are in accordance with tumor growth, which follow the order of SOX8 over‐expression, vector, SOX8 over‐expression combined with GOLPH3 knockdown, and GOLPH3 knockdown in succession (B and C). HE staining and metastatic tumor counting suggests that, SOX8 over‐expression enhances metastasis formation in lungs (D). In contrast, GOLPH3 knockdown markedly reduces metastasis number in lungs (E). Immunochemical staining is carried out to analyze the protein levels of SOX8, GOLPH3, and Ki67 in xenografts (F). The results show that, GOLPH3 expression is markedly up‐regulated in the SOX8 over‐expression group, and that GOLPH3 staining is notably increased in SOX8 over‐expression combined with GOLPH3 knockdown group, compared with that in GOLPH3 knockdown group. Silencing GOLPH3 expression dramatically reduces Ki67 expression relative to control groups. However, SOX8 over‐expression up‐regulates Ki67 expression level. Interestingly, Ki67 expression is up‐regulated in the SOX8 over‐expression combined with GOLPH3 knockdown group, compared with that in GOLPH3 knockdown group

For confirming the role of SOX8 in regulating the growth of TSCC tumor in vivo, the TSCC xenograft nude mouse model was constructed. Each mouse was injected with SCC25 cells that had vector plasmids or stably expressed SOX8 plasmids. In addition, GOLPH3 shRNA plasmids were transfected to the SOX8‐overexpressing cells, which were then administered to nude mice for establishing the rescue model. Growth of tumor was observed for four weeks continuously. Our results suggested that, the SOX8‐overexpressing SCC25 xenografts grew at a faster rate compared with those under control vector treatment. In contrast, SCC25 xenografts with knockdown ofGOLPH3 grew slower (Figure 5A). Moreover SCC25 xenografts with GOLPH3 knockdown grew slowly with regards to tumor weight and size compared with those in control as well as SOX8‐overexpression group (Figure 5B), but xenografts with SOX8 over‐expression and GOLPH3 knockdown showed fast growth with regards to tumor weight and size, relative to those showing knockdown of GOLPH3, together with those under control vector treatment (Figure 5B and C). Collectively, these results indicated that SOX8 promoted TSCC tumor growth via GOLPH3.

Tail vein injection was performed to establish the animal model with lung metastasis, which was confirmed through HE staining. It was found that SOX8 over‐expression enhanced lung metastases (Figure 5D). Nonetheless, GOLPH3 knockdown evidently reduced lung metastases compared with those in non‐sense shRNA‐treated control samples. Besides, SOX8 over‐expression within GOLPH3 knockdown SCC25 cells boosted lung metastases in comparison with those in GOLPH3 knockdown SCC25 cells samples. Of course, the samples with only SOX8 over‐expression SCC25 cells grew faster than all others (Figure 5E).

In addition, SOX8, GOLPH3, and Ki67 protein levels were analyzed within xenografts through immunochemical staining. According to our results, GOLPH3 was up‐regulated in the SOX8‐overexpression group (Figure 5F), and GOLPH3 staining was notably increased in the SOX8 over‐expression combined with GOLPH3 knockdown group, relative to that in GOLPH3 knockdown group. Also, effects of GOLPH3 knockdown and SOX8 over‐expression on Ki67 (a vital factor indicating tumor growth) expression in xenografts was investigated. GOLPH3 silencing within the nude mice model of TSCC evidently down‐regulated Ki67 expression, compared with that in control groups (Figure 5F). However, SOX8 over‐expression can up‐regulate Ki67 expression(Figure 5F). Interestingly, in the SOX8 over‐expression combined with GOLPH3 knockdown group, Ki67 expression was prominently up‐regulated, compared with that in GOLPH3 knockdown group (Figure 5F). Collectively, the above findings in vivo conformed to those results observed in vitro, which validated that SOX8 exerted the regulatory part for the metastasis and growth of TSCC tumor by controlling GOLPH3 expression.

### 2.6 SOX8 interacted with TFAP2A to regulate GOLPH3 expression and TSCC cell growth

**FIGURE 6:**
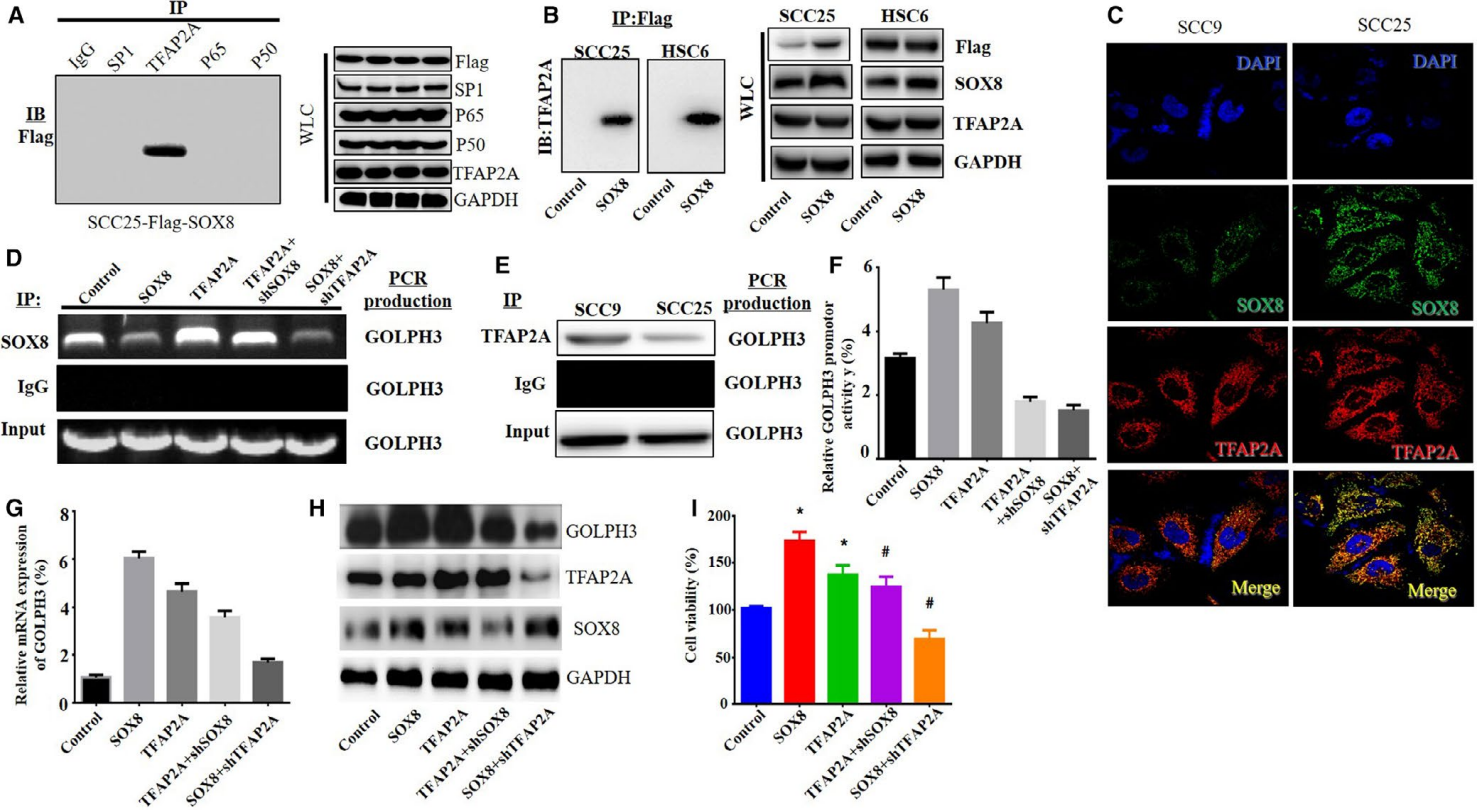
SOX8 interacts with TFAP2A to regulate GOLPH3 expression and tongue squamous cell carcinoma (TSCC) cell growth. Co‐immunoprecipitation assay is carried out to test 4 predicted GOLPH3 promoter‐binding proteins, namely, p65, p50, SP1, and TFAP2A, using specific antibodies against each of these proteins in the flag‐SOX8‐overexpressing SCC25 and HSC6 cells. Surprisingly, SOX8 is knocked down via the anti‐TFAP2A antibodies (A). Besides, the SOX8‐TFAP2A interaction is also validated in the other co‐immunoprecipitation assay that uses the SOX8‐specific antibodies (B). Additionally, dual‐immunofluorescence assay is used for further analyzing TFAP2A and SOX8 expression as well as the sub‐cellular localization within SCC9 as well as SCC25 cells. SOX8 (green) is expressed both within the cytoplasm and nucleus, while TFAP2A (red) expression is mainly detected in cytoplasm. The TFAP2A‐SOX8 co‐localization is detected in the nucleus and cytoplasm (yellow). On the other hand, ChIP assay confirms that TFAP2A binds onto GOLPH3 promoter in SCC9 and SCC25 cells (E). Furthermore, the over‐expression of TFAP2A enhances SOX8 binding onto the GOLPH3 promoter. By contrast, the knockdown of TFAP2A weakens SOX8 binding onto GOLPH3 promoter within the SCC25 cells with SOX8 over‐expression. Knockdown of SOX8 weakens SOX8 binding onto the GOLPH3 promoter within SCC25 cells with TFAP2A over‐expression (D). Moreover, luciferase reporter gene assay is carried out, which suggests that TFAP2A over‐expression enhances activity of GOLPH3 promoter, whereas knockdown of TFAP2A suppresses activity of GOLPH3 promoter. Additionally, only SOX8 knockdown or TFAP2A knockdown reduces the GOLPH3 promoter activity in SCC25 cells with TFAP2A or SOX8 over‐expression, respectively (F). Western Blotting (H) and RT‐PCR (G) suggest that, TFAP2A over‐expression up‐regulates GOLPH3 mRNA and protein expression, whereas the expression of GOLPH3 is down‐regulated following TFAP2A knockdown, even in SOX8‐overexpressing SCC25 cells. Finally, CCK8 assay indicates that TFAP2A knockdown inhibits the increased TSCC cell growth induced by SOX8 over‐expression, while over‐expression of TFAP2A partially rescues the inhibition of cell growth mediated by SOX8 knockdown (I)

Subsequently, this study examined whether SOX8 bound to the GOLPH3 promoter via the interaction with other transcriptional factors (such as p65, p50, SP1, and TFAP2A). Few studies had performed co‐immunoprecipitation assay to test our predicted GOLPH3 promoter‐binding proteins, namely, p65, p50, SP1, and TFAP2A. Surprisingly, anti‐TFAP2A antibodies knocked down SOX8 (Figure 6A). Additionally, SOX8‐TFAP2A interaction had been repeated in the other co‐immunoprecipitation experiment using the SOX8‐specific antibodies (Figure 6B). Besides, the dual‐immunofluorescence assay was carried out for testing SOX8 and TFAP2A expression as well as their sub‐cellular localization within SCC9 as well as SCC25 cells. According to Figure 6C, SOX8 (green) was located in the nucleus and cytoplasm, whereas TFAP2A (red) was located within nucleus. TFAP2A‐SOX8 co‐localization was observed in both cytoplasm and nucleus (yellow).Furthermore, the over‐expression of TFAP2A and SOX8 promoted SOX8 binding onto the GOLPH3 promoter (Figure 6D, lane2, and lane4, respectively). By contrast, knockdown of TFAP2A moderated SOX8 binding onto the promoter of GOLPH3 within SCC25 cells with SOX8 over‐expression (Figure 6D, lane3). SOX8 knockdown moderated SOX8 binding onto the GOLPH3 promoter in SCC25 cells with TFAP2A over‐expression (Figure 6D, lane5). TFAP2A binding ontopromoter of GOLPH3 in SCC9 and SCC25cells was also validated through ChIP assay (Figure 6E). On the other hand, luciferase reporter gene assay revealed that, TFAP2A over‐expression promoted activity of GOLPH3 promoter, while that was restrained by knockdown of TFAP2A (Figure 6F). Additionally, either SOX8 knockdown or TFAP2A knockdown inhibited the GOLPH3 promoter activity in SCC25 cells with TFAP2A over‐expression or SOX8 over‐expression, respectively (Figure 6F). For examining TFAP2A effect on GOLPH3 expression, RT‐PCR, and western blotting were carried out. Figure 6G and H suggested that, TFAP2A over‐expression up‐regulated GOLPH3 mRNA and protein expression, while the expression of GOLPH3 was down‐regulated following TFAP2A knockdown, even in SCC25 cells with SOX8 over‐expression. Finally, TFAP2A effect on the viability of cells was also examined through the CCK8 assay, which revealed that the knockdown of TFAP2A suppressed the increased TSCC cell growth induced by SOX8 over‐expression, while the over‐expression of TFAP2A partially restored the suppression of cell growth mediated by SOX8 knockdown (Figure 6I). The above findings suggested that, the interaction between SOX8 and TFAP2Aregulated GOLPH3 promoter activity, gene expression, and the proliferation of TSCC cells.

### 2.7 SOX8 was positively correlated with GOLPH3 in TSCC cell lines and tumor tissues

**FIGURE 7:**
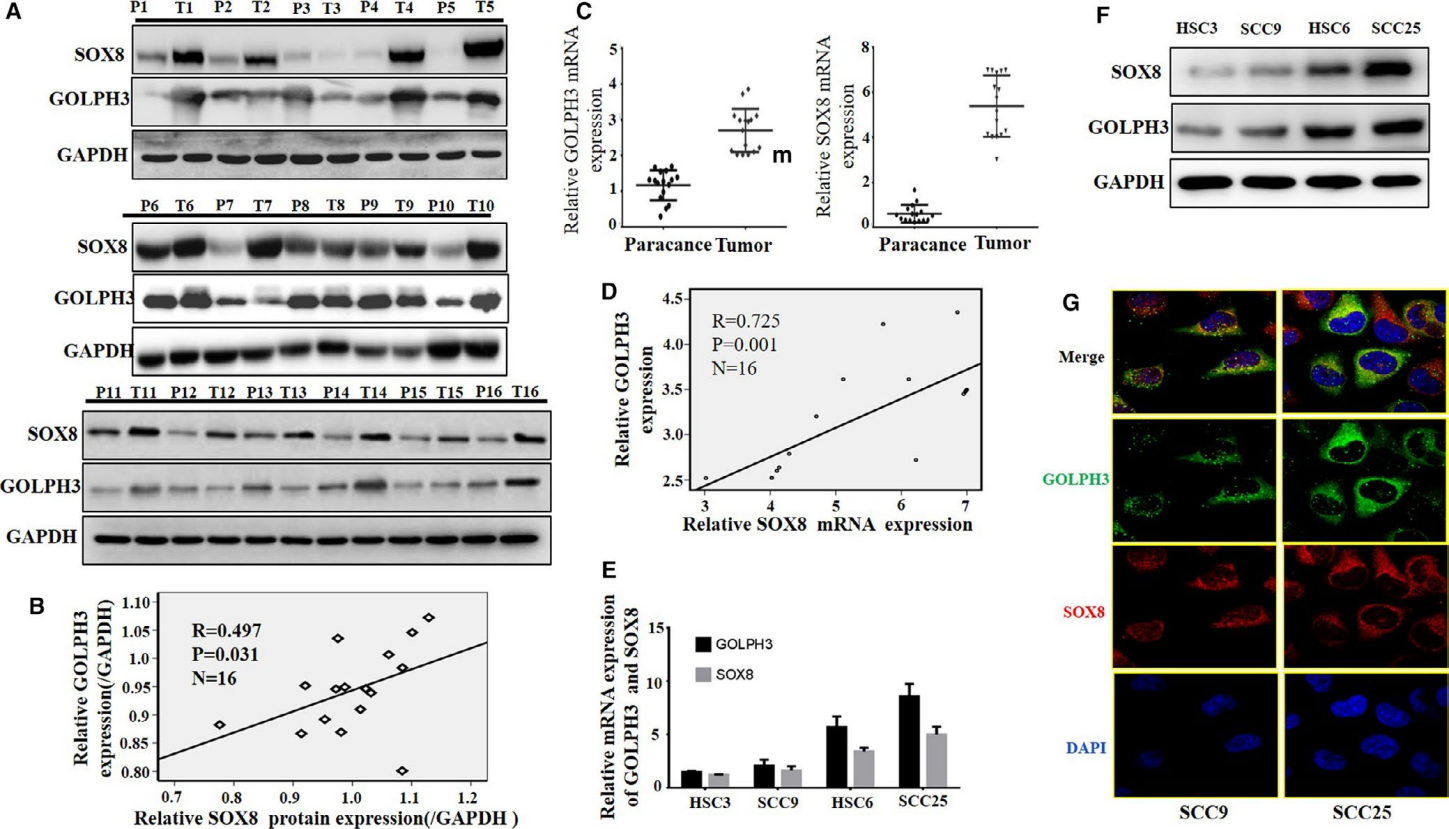
SOX8 shows positive correlation with GOLPH3, and these two are highly expressed in tongue squamous cell carcinoma (TSCC) cell lines and tumor tissues. By immunoblotting analysis, the SOX8 and GOLPH3 protein expression is evidently up‐regulated within TSCC tissues compared with that in matched para‐cancer counterparts (A). Besides, the SOX8 protein expression shows positive correlation the GOLPH3 protein expression (Pearson correlation test; n = 16, *r* = .497, *P* = .031) (B). Furthermore, our findings show that, both SOX8 and GOLPH3 mRNA levels are obviously increased within TSCC tumor tissues relative to those within matched non‐cancer counterparts (C). In addition, SOX8 mRNA level shows positive correlation with the GOLPH3 mRNA level (D). Finally, the SOX8 and GOLPH3 protein and mRNA expression is analyzed within TSCC cell lines via western blotting and RT‐PCR, respectively. According to our findings, SOX8 expression is positively correlated with GOLPH3 expression (E and F). Analysis of subcellular localization via immunofluorescence indicates that SOX8 and GOLPH3 are mainly expressed in the cytoplasm. The above findings further demonstrate the regulatory effect of SOX8 on GOLPH3 within TSCC (G)

Sixteen pairs of tumor tissues together with matched para‐cancer counterparts were collected from TSCC patients. According to immunoblotting analysis, SOX8 and GOLPH3 protein expression was dramatically up‐regulated within TSCC tissues compared with that in matched para‐cancer counterparts (Figure 7A). In addition, SOX8 protein expression was found to show positive correlation with GOLPH3 protein expression (Pearson correlation test; n = 16, *r* = .497, *P* = .031) (Figure 7B). Furthermore, the SOX8 and GOLPH3 mRNA levels were obviously up‐regulated within TSCC tumor tissues relative to those within matched non‐cancer counterparts (Figure 7C). SOX8 mRNA expression was positively correlated with the GOLPH3 mRNA expression (Figure 7D). In addition, RT‐PCR and western blotting obtained similar results in TSCC cell lines (SCC9, SCC25, HSC3, and HSC6; Figure 7E and F). Immunofluorescence indicated that the subcellular localization of SOX8 and GOLPH3 was mainly observed in the cytoplasm (Figure 7G).

### 2.8 High SOX8 and GOLPH3 expression was related to dismal clinical outcomes for TSCC patients

**FIGURE 8:**
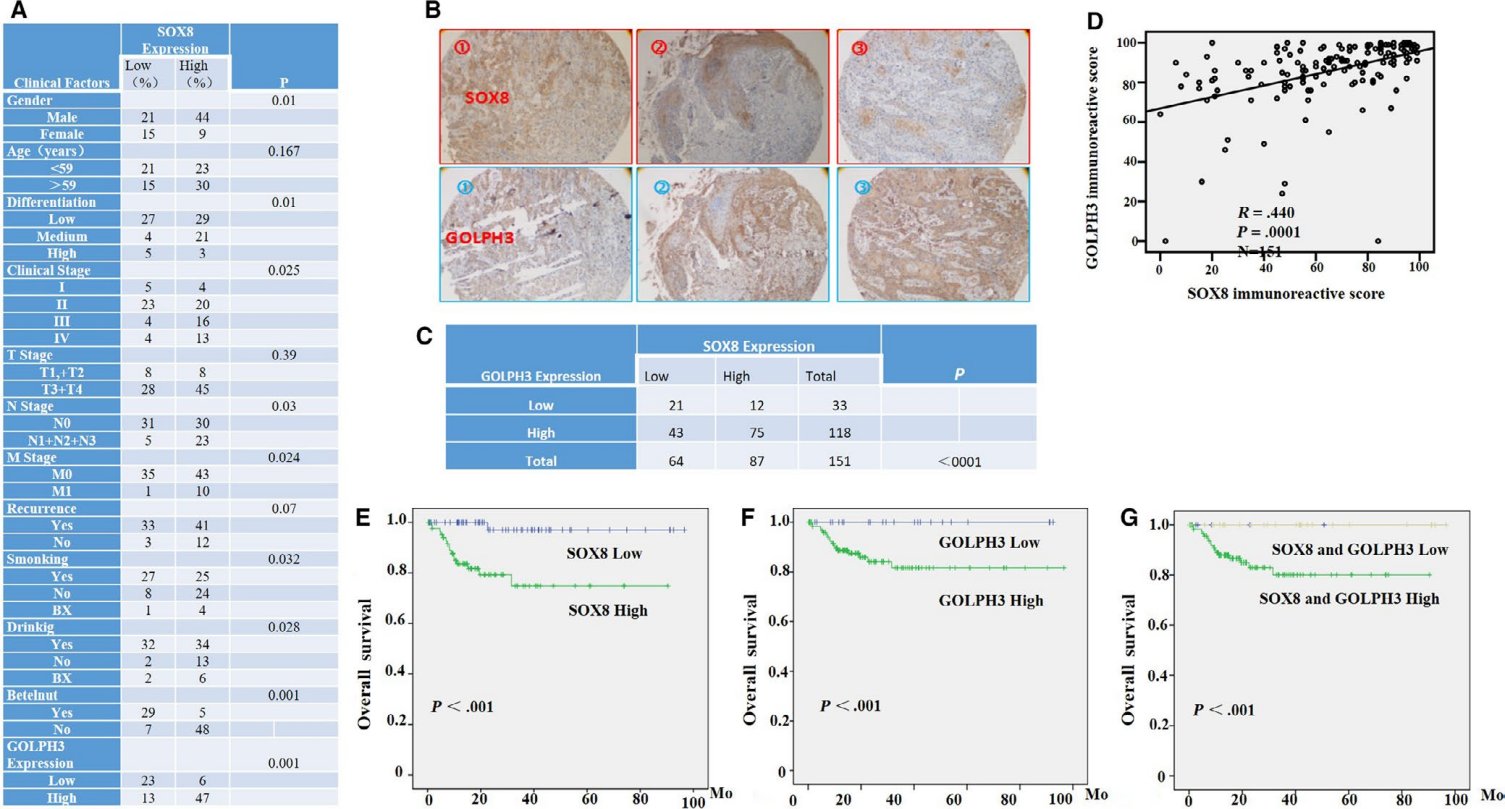
High SOX8 and GOLPH3 expression Levels are related to dismal clinical results for tongue squamous cell carcinoma (TSCC) patients. Tissue microarrays (n = 89) are used to investigate the influence of up‐regulated SOX8 and GOLPH3 expression on the clinical results for TSCC patients. The relationships of SOX8 level with clinicopathological parameters from the 89 TSCC patients are displayed in (A). IHC analysis shows the representative results of SOX8 and GOLPH3 in tumor tissues from three different patients (B). High expression of SOX8 and GOLPH3 is also detected, which is positively correlated within TSCC tissues (C, D). Moreover patients that have up‐regulated SOX8 level are associated with reduced overall survival (OS) compared with patients having low SOX8 level (E), and this is also true regarding the GOLPH3 level (F). In addition, the OS for cases with up‐regulated SOX8 and GOLPH3 expression is dramatically reduced compared with that for patients with low SOX8 and GOLPH3 expression (G)

SOX8 and GOLPH3 expression within TSCC tissue microarrays from 89 patients with complete follow‐up data was investigated. Our results revealed high SOX8 and GOLPH3 expression, which showed positive correlation within TSCC tissues (Figure 8A–C). Figure 8D displays the relationships of SOX8 expression with the clinicopathological parameters from89 TSCC patients. High SOX8 expression was found to be markedly related to the pN stage (*P* = .003), pM stage (*P* = .024), Betel nut‐chewing history (*P* = .0001), smoking (*P* = .032), and drinking (*P* = .028). However, SOX8 expression was not correlated with the age of patient (*P* = .167), pT stage (*P* = .39), recurrence (*P* = .077), as well as differentiation (*P* = .062). Moreover patients that had SOX8 up‐regulation showed reduced overall survival (OS) compared with patients having SOX8 down‐regulation (Figure 8E), and this was true for GOLPH3 expression (Figure 8F). In addition, the OS for patients with up‐regulated SOX8 and GOLPH3 expression was markedly reduced compared with that for patients who showed low SOX8 and GOLPH3 expression (Figure 8G).

## 3 DISCUSSION

GOLPH3 has been proved to be the oncoprotein, which participates in the development of a variety of cancer types.^6^ GOLPH3 is indicated in recent studies to trigger the PI3K/AKT/mTOR signal transduction pathway via mTORC1 and mTORC2 phosphorylation, resulting in cell proliferation.^30^ In addition, GOLPH3 is considered as the new candidate target of many tumor therapies.^9^, ^10^, ^15^, ^16^, ^31^ Accumulating evidence has shown that there may be differential expression of upstream factor, which particularly binds to GOLPH3 promoter for regulating the expression of GOLPH3. This study was the first to identify thatSOX8 was a protein binding to GOLPH3 promoter within the TSCC cell lines. According to our results, SOX8regulated GOLPH3 expression at transcriptional level, and SOX8‐mediated cell growth, migration, and invasion through GOLPH3. Moreover it was found that SOX8 enhanced the growth of xenograft and the formation of lung metastases in vivo through the GOLPH3 signal transduction pathway. Additionally, TFAP2A was confirmed as the binding factor between SOX8 GOLPH3 promoter‐binding region. The above findings had revealed that, there was interaction between SOX8 and TFAP2A, which modulated the progression and growth of TSCC tumor through GOLPH3 expression. SOX8, one of the E subgroup members for SOX genes, participates in some human diseases. The SOX8 homologs are shown to be over‐expressed within the embryonic as well as adult brain. Few reports have examined SOX8 in tumors. For instance, Zhang S et al found that, SOX8 was markedly up‐regulated in HCC, which promoted HCC cancer cell proliferation.^24^ Additionally, the low‐grade diffuse astrocytomas and oligodendroglial tumors are associated with particularly high SOX8 expression.^23^ However, it remains unclear about the expression and function of SOX8 in TSCC. According to our results, the expression of SOX8 was usually up‐regulated within human TSCC cells, compared with the immortalized cells. SOX8 down‐regulation inhibited the invasion, migration and proliferation of TSCC cells, which were enhanced by SOX8 up‐regulation. Moreover, it was also discovered in this study that, SOX8 knockdown‐mediated cell growth, migration, and invasion inhibition was restored through the over‐expression of GOLPH3. Additionally, this study also confirmed SOX8 effects on enhancing tumor progression and growth in vivo, and GOLPH3 participated in the biological process of SOX8 in TSCC. According to the results of double staining immunohistochemistry, there was high SOX8 and GOLPH3 expression in tumor tissues from TSCC patients relative to adjacent non‐cancer counterparts. K‐M analysis revealed that high SOX8 expression was related to the poorer prognostic outcomes and more aggressive tumor progression, indicating that SOX8 increased the TSCC susceptibility. However, the reason responsible for SOX8 up‐regulation in TSCC remains unclear so far. It is shown in another study^32^ that, the methylation status of SOX8 gene is increased with the different cancer clinical stages in cervical curettage from cervical cancer patients, compared with those from normal individuals. In addition, SOX8 gene exhibits high methylation level in cancerous tissues, but not in normal tissues. The abnormal gene promoter methylation may be one of the causes of SOX8 up‐regulation in TSCC, which should be investigated in our future studies.

SOX9 and SOX10 are the other two members of the SOX‐E family, which have been reported to participate in tumorigenesis.^21^, ^26^, ^27^ However, neither SOX9 nor SOX10 regulates GOLPH3transcription and translationin TSCC cells. SOX8 is also involved in transcriptional regulation, neural and chondrogenic differentiation, biogenesis of the mammalian nervous system, and testis development.^20^, ^33^, ^34^ Despite the increasing knowledge regarding SOX8, it is still unclear about the contribution of SOX8 to mammalian cell development as well as the functional characterizations. The DNA‐dependent dimerization in sites that bear the (A/T)(A/T)CAA(A/T)G consensus sequence can be mainly ascribed to the SOX‐E family.^35^ This study potently suggested that, SOX8 bound toGOLPH3 promoter, while regulating GOLPH3 transcription, translation and proliferation of TSCC cells. Nonetheless, it is still unclear about whether SOX8 DNA‐binding domain is involved in its binding with the GOLPH3 promoter. Our findings revealed a new role of SOX8, but its role in regulating tumorigenesis‐related genes should be further examined.

Previous studies illuminate that GOLPH3 is a oncogene, which facilitates the occurrence and development of multiple signal transduction pathways and cell processes within some tumor types.^7^, ^9^, ^11^, ^15^, ^30^ It was pointed out in our study that, SOX8 regulated the biological behaviors and prognosis of TSCC via the downstream GOLPH3signal transduction pathways. Moreover SOX8 up‐expression was found to enhance the expression of GOLPH3, which thereby induced the activation of PI3K/AKT/mTOR signal pathway andup‐regulatedFOXO1 expression, and this in turn promoted TSCC cell growth. These findings were in line with previous studies.^6^, ^11^, ^36^ Increasing evidence indicates that EMTplays a key role in tumor invasion and metastases.^9^ According to prior work, β‐catenin, E‐cadherin, N‐cadherin, Vimentin, Snail, and c‐Myc are the downstream effectors of the GOLPH3 signaling.^9^, ^15^ GOLPH3 knockdown down‐regulated the expression of E‐cadherin and Snail. Based on our findings, SOX8 modulated the invasion and migration of TSCC cells bot*in vivo* and *in vitro*throughGOLPH3 and β‐catenin, N‐cadherin, E‐cadherin, c‐Myc, Snail, and Vimentin. Collectively, these results suggested that SOX8 regulated the TSCC biological behaviors and prognosis through the downstream GOLPH3‐regulated signal transduction pathways.

Is the expression of SOX8 and GOLPH3 also closely correlated in the control cell line? This hypothesis may suggest the changes in gene functions during the malignant transformation of cells. However, answering this question requires a new elegant‐designed study to confirm.

To sum up, findings in this study demonstrate that GOLPH3 facilitates the proliferation, migration, and invasion of TSCC cells. Besides, high GOLPH3 expression is a driver and hallmark of TSCC progression. Furthermore, SOX8 is recognized to be the new protein binding to GOLPH3 promoter in TSCC cells, which is also verified to be the factor of up‐regulating GOLPH3 signaling. In addition, the interaction between SOX8 and TFAP2A is found to modulate GOLPH3 expression and its promoter activity. Finally, our study confirms high SOX8 expression in TSCC tumor tissues compared with that in adjacent non‐tumor counterparts, which shows positive correlation with GOLPH3 over‐expression and poor survival. Our data suggest that the SOX8/GOLPH3 pathway may serve as the candidate treatment target for TSCC patients.

## 4 MATERIALS AND METHODS

### 4.1 Cells as well as antibodies

TSCC cells (including SCC25, SCC9, HSC3, and HSC6) were provided by American Type Culture Collection (ATCC, Rockville, MD, USA). Then, all cells were cultivated within the Dulbecco's Modified Eagle's Medium (Invitrogen, Carlsbad, CA) containing 10% fetal bovine serum (FBS). In addition, the human oral epithelial NOK cells were cultivated within KFS (Gibco BRL, Grand Island, NY) containing 10% FBS. Subsequently, all the cells had been kept at 37°C under humid atmosphere containing 5% CO_2_.

Meanwhile, the TSCC tissue specimens had been extracted based on the Tumor Bankof Sun Yat‐sen UniversityCancer Center (Guangzhou, China) from July 2002 to December 2016. Our study protocol had gained approval from the Ethics Committee of the Sun Yat‐sen University Cancer Center. Each patient submitted the informed consent for participation.

### 4.2 Antibodies and reagents

MTS had been provided by the Qiagen (Hilden, Germany). SOX8 antibodies that were used in immunofluorescence staining, ChIP and Western Blotting were provided by Cell Signaling Technology (12943s), Merck Millipore (MAB377), and Sigma (sab4301175), respectively. The remaining antibodies had been provided by Cell Signaling Technology (Danvers, MA).

### 4.3 Assay for streptavidin‐agarose pull‐down

The streptavidin‐agarose pull‐down experiment was carried out according to previous description to analyze the proteins binding to GOLPH3 promoter.^37^, ^38^ Firstly, extracts of nuclear protein (0.5 mg) isolated based on TSCC cancer cells would be subjected to incubation with the biotin‐conjugated double strand DNA probes (20 µg)matched with the nucleotides from −585 to +1 in the promoter region of GOLPH3 (Sigma‐Aldrich), together with the agarose‐streptavidin beads (200 µL, Sigma‐Aldrich) overnight under 4°C. Then, the mixture would be subjected to 2 hours of incubation under shaking under the ambient temperature. Later, the beads were centrifuged to make pellets and rinsed using PBS. Afterwards, the 10% PAGE gel was utilized to separate those binding proteins for western blotting assay.

### 4.4 Identification of GOLPH3 promoter‐binding proteins

Proteins bound onto GOLPH3 promoter were pulled down and analyzed by mass spectrometry (MS). First of all, the separated bound proteins stained with silver were used (Beyotime, Shanghai, China), then potential protein bands were digested by MS‐grade trypsin solution (Promega, Madison, WI) following alkylation and reduction, and then MS was performed. For those proteins of interest, their identifications were validated by referring to the existing software and databases. We finally identified SOX8 protein based on mass spectrometry (Figure S1).

### 4.5 Transient transfection

For over‐expressing SOX8 and TFAP2A within the TSCC cell lines, all cell lines were subjected to transfection using pcDNA3.1‐SOX8, pcDNA3.1‐SOX9, pcDNA3.1‐SOX10, pcDNA3.1‐TFAP2A, and the control vector plasmid pcDNA3.1‐LacZ using Lipofectamine 3000 (Invitrogen, Carlsbad, CA). In addition, for inhibiting the expression of SOX8, TFAP2A, SOX9, SOX10, and GOLPH3, the TSCC cell lines would be subjected to transfection with short‐hairpin RNA specific to SOX8 (shRNA, 5'‐GGC TAC ACG TCT CCA ACA T‐3'and 5'‐GCG GCA AAT GTT CGG GCA A‐3'), shRNA specific to TFAP2A (5'‐GAG CCU GUG UAU GGC AAU ATT‐3' and 5'‐GCA CGU GUA AUG ACA AAU ATT‐3'), shRNA specific to SOX9(5'‐GCU GCA UGU CUC UAA UAU UTT‐3' and 5'‐GCC ACA CAC UCA AGA CUA UTT‐3'), shRNA specific to SOX10(5'‐GCU GCA UGU CUC UAA UAU UTT‐3'and 5'‐GCC ACA CAC UCA AGA CUA UTT‐3'), siRNA specific to GOLPH3 (5'‐GCU GCA UGU CUC UAA UAU UTT‐3' and 5'‐GCC ACA CAC UCA AGA CUA UTT‐3'), short‐hairpin RNA specific to GOLPH3 (shRNA, 5'‐GGC TAC ACG TCT CCA ACA T‐3' and 5'‐GCG GCA AAT GTT CGG GCA A‐3'), and siRNA specific to TFAP2A (5'‐GGA CCA GUC UGU CAU UAA ATT‐3'). The siRNAs were provided by Shanghai GenePharma Co. (Shanghai, China).

### 4.6 Chromatin immunoprecipitation (ChIP) experiment

ChIP assay was carried out in accordance with our previous description.^37^ Briefly, all cells had been subjected to 1% formaldehyde fixation, and then 1.4 mol/L glycine (0.1 mL/mL culture) was added to quench the cross‐linking. Afterwards, each sample was subjected to on‐ice sonication for shearing DNA to fragments at 300‐1000 bp. Later, all total cell lysates were divided into three equal parts, among which one part was utilized to be DNA input control, one was used for immunoprecipitation using anti‐SOX8 antibodies, while one was treated with non‐immunologic rabbit IgG (Cell Signaling Technology, Danvers, MA). Then, the spin columns (Qiagen, Hilden, Germany) were used to purify the DNA fragments, and the GOLPH3 promoter region was amplified by PCR using the primer pairs below: 5'‐TGGCCCCTCCCTCGGGTTAC‐3' (forward), and 5'‐TGAAG GGGCAGGACGG.

GTGC‐3' (reverse). Moreover, 2% agarose gel electrophoresis was carried out to resolve those PCR products, followed by visualization using Gel‐Red staining.

### 4.7 Cell transfection and lentiviral construction

For producing the clones with stable SOX8 and GOLPH3 over‐expression, the empty lentiviral control vector, or the lentiviral vector that encoded the full‐length GOLPH3 and SOX8 genes was transfected into SCC9 and HSC3 cell lines. Afterwards, the stable clones would be screened at 2 weeks later by applying selection pressure using 10 µg/mL puromycin, and SOX8 and GOLPH3 expression was examined through qRT‐PCR and Western Blotting.

The U6‐sh‐SOX8‐puromycin‐EGFP‐IRES vectors had been provided by Genechem Co. Ltd (Shanghai, China), which were utilized for SOX8 knockdown. The lentiviral vector that contained the non‐silencing shRNA was would be utilized to be negative control. SCC25 as well as HSC6 cell lines were then transfected with negative control vector or lentiviral vectors that encoded the typical shRNA sequences for SOX8. Later, the RNA interference efficiency was detected through western blotting and qRT‐PCR.

### 4.8 Luciferase reporter gene assay

The fragment that contained the core GOLPH3 promoter region (−585 to +1) had been inserted at between HindIII and SacI sites in firefly luciferase vector pGL4.10 (Promega, Madison, WI), with the control Renilla luciferase reporter vector pRL‐TK being utilized to be the control. Afterwards, cells (2 × 10^4^ cells/well) had been grown into the 96‐well plates. When reaching 50% cell confluence, the SCC25, SCC9, HSC3, and HSC6 cell lines that had stable SOX8 over‐expression or knockdown were transfected with pRL‐TK or pGL4.10‐GOLPH3(585)using Lipofectamine 3000 (5:1 ratio). At 48 hours following transfection, the Dual‐Luciferase® Reporter Assay System (Promega, Madison, WI) was used to carry out the dual‐luciferase reporter gene assay.

### 4.9 Assay to test viability of cells

TSCC cells had been grown at 5 × 10^3^ cells/well within the 96‐well plates, and MTS assay (Promega, Madison, WI) was conducted 72 hours after cell culture to assess the cell viability. Sample absorbance in each well at the wavelength of 490 nm would be determined by the microplate reader. Five replicates per trial were performed for all experiments, and a total of 3 independent trials were carried out, so as to calculate the mean cell viability percentage.

### 4.10 Colony‐forming analysis

TSCC cells had been grown at 400 cells/well within the six‐well plates, followed by 10‐14 days of culture. Afterwards, 1% crystal violet was used to stain the colonies, and the stained ones were counted. Three independent trials had been carried out for all experiments.

### 4.11 Transwell invasion and wound healing assays

Transwell invasion and wound healing assays were carried out for determining the TSCC cell invasion and mobility, respectively, under the conditions of SOX8 knockdown and overexpression. TSCC cells that had stable SOX8 knockdown or over‐expression or cells under control vector treatment had been cultivated within the 6‐well plates until reaching cell confluence. Then, the pipette tip (10‐μL) was used to scratch the cell layers. At 0 and 24 hours following scratching, the cell wound‐healing images were obtained. All samples were examined for three times. Then, the BD BioCoat Matrigel Invasion Chambers (Becton Dickinson, Franklin Lakes, NJ) was adopted for testing the cell invasion according to manufacturer protocol. Five fields of view (FOV) were selected for each sample, and light microscope was employed for counting.

### 4.12 Invasion and migration analyses

The Boyden chambers that contained the 24‐well Transwell plates (BD Biosciences, with the pore size of 8 mm) were used in Transwell assay for evaluating cell invasion and migration capacities. Each experiment was carried out for twice and then repeated thrice. In migration assay, 1.0 × 10^5^ cells were seeded into the inserts of cell culture without an extracellular matrix coating on their membranes within the serum‐free DMEM (200 μL). In addition, the bottom chamber was added with the DMEM medium supplemented with 20% FBS. After approximately 24 hours of incubation, the cells located on bottom filter surface had been subjected to fixation and staining following incubation for 24 hours, which were later detected by the microscope. In invasion assay, Matrigel (60 μL, 1:10, BD Biosciences) was used to coat the membrane. Following 4 hours of Matrigel solidification at 37°C, 3.0 × 10^5^ cells were seeded into the inserts of cell culture within the serum‐free DMEM (200 μL), whereas DMEM supplemented with 20% FBS was added into the bottom chamber. Moreover the Boyden chamber would be subjected to 24 hours of incubation at 37°C under 5% CO_2_ conditions. Cells were then fixed, stained, and examined microscopically.

### 4.13 Western blotting assay

The lentiviral constructs were used to transfect the TSCC cell lines; 2 weeks later, the cells were selected via selection pressure using puromycin. The RIPA lysis buffer (Beyotime Biotechnology, Shanghai, China) and Complete Lysis‐M reagent (Roche, USA) were used to prepare the nuclear extracts or whole cell lysates. Later, the protein concentrations were measured through the BCA assay (ThermoFisher Scientific, Waltham, MA). Afterwards, proteins would be isolated through 10% SDS‐PAGE, followed by transfer to the PVDF membranes. The anti‐SOX8, anti‐GOLPH3, anti‐TFAP2A, anti‐Histone3, anti‐p‐PI3K, anti‐PI3K, anti‐p‐AKT, anti‐AKT, anti‐p‐GSK3β, anti‐GSK3β, anti‐FOXO1, anti‐p‐FOXO1, anti‐β‐catenin, anti‐N‐cadherin, anti‐E‐cadherin, anti‐Snail, anti‐Vimentin, and anti‐GAPDH antibodies were provided by Cell Signaling Technology (Danvers, MA). Meanwhile, the anti‐c‐Myc rabbit antibodies were provided by Abcam (Cambridge, UK).

### 4.14 Real‐time PCR (qPCR)

The TRIZOL Reagent (Invitrogen, Carlsbad, CA) was used to isolate the total RNA according to the manufacturer's protocols. The ReverTra Ace qPCR RT Master Mix (Toyobo, Japan) was utilized for cDNA synthesis. qPCR was carried out by SYBR Green PCR master mix (Toyobo, Osaka, Japan), while the Bio‐Rad CFX96 system was employed to detect products, and Bio‐Rad Manager software was utilized for product analysis. The relative expression of tested genes compared with SOX8 or GOLPH3 expression was calculated by the 2^−ΔCT^ method (ΔCT = CT_SOX8_ or CT_GOLPH3_ ‐ CT_GAPDH_), which was then standardized based on relative levels in control cells. All samples were examined for three times. The primers used in this study were provided by GeneCopoeia.

### 4.15 Co‐immunoprecipitation tests

Equivalent extracts of nuclear protein collected based on variouscell types were subjected to specific antibody incubation. Later, agarose‐labeled protein‐A/G beads (Merck Millipore, Billerica, MA) had been added, followed by overnight incubation of the mixture at 4°C. Then, the beads were washed by the cold phosphate buffered saline (PBS), and added with the loading buffer before being boiled. Western Blotting was carried out to detect proteins containing within the supernatant.

### 4.16 Tumor model in vivo as well as tissue treatment

Each animal experiment was carried out according to Institutional Ethical Guidelines for Animal Experiments formulated via Sun Yat‐sen University, together with the Guide for the Care and Use of Laboratory Animals. Each animal was provided by Shanghai Institutes for Biological Sciences (Shanghai, China). 2 × 10^6^ SCC25 cells that had stable SOX8 expression was injected subcutaneously into four groups of five 4‐week‐old female nude mice (with the weight of 16‐18 g). The length (a) and width (b) of all tumors were determined by the calipers, whereas tumor volume (V) was computed according to the following formula: V = 1/2(a × b × b). The SOX8 and GOLPH3 expression in tumor tissues from TSCC patients and xenograft tissues from nude mice was determined through IHC staining. Then, tumor sections were deparaffinized, blocked, retrieved with antigen, and incubated with the SOX8‐specific antibodies (1:50, Sigma), Ki67‐specific antibodies (1:50, Sigma), or the GOLPH3‐specific antibodies (1:100, Nouvs Biologicals, Littleton, CO) overnight under the temperature of 4°C within the humid chamber. Afterwards, tumor sections would be washed, incubated by the horseradish peroxidase (HRP)‐conjugated anti‐goat antibodies under ambient temperature for 30 minutes, then subjected to color development using the chromogenic substrate 3,5‐diaminobenzidine (DAB),and counter‐stained with Mayer's hematoxylin. To calculate the IHC score, the stained tumor cell percentage (0‐100%) was used to multiply by staining intensity (0, 1, 2, or 3), which yielded the score of 0‐300.

### 4.17 Metastasis models

The lung metastasis model was adopted. To establish a lung metastasis model, indicated cells had been collected and rinsed by 1 × PBS for twice, followed by suspension into PBS. About 5 × 10^6^ TSCC cells containing within 150 μL PBS were injected to the male athymic mice (5weeks old) through the tail veins. Then, the mice were killed after observed for 7‐8 weeks to count, excise and embed the tumor nodules on lung surface in paraffin.

### 4.18 Statistical methods

Values were expressed in the manner of means ± standard deviations for three independent experiments at least. The SPSS 16.0 (SPSS Inc; Chicago, IL) was adopted for all statistical analyses. A difference of *P* < .05 would be deemed to be of statistical significance.

## CONFLICTS OF INTEREST

The authors declare no competing financial interest.

## AUTHORS' CONTRIBUTIONS

DS, SC, and HL performed most of the experiments, analyzed data, and wrote the manuscript. XL performed some experiments. ZY, XZ, and WC collected the specimen. ZC, JC, and YZ reviewed and edited the manuscript. DS and MS designed the experiments and edited the manuscript. All authors read and approved the final manuscript.

## Supporting information

Supplementary Material

## Data Availability

All data included in this study are available upon request by contact with the corresponding author.
