# Supplementary figures and images for: High SOX8 expression promotes tumor growth and predicts poor prognosis through GOLPH3 signaling in tongue squamous cell carcinoma
Source: Cancer Med. 2020 Apr 19;9(12):4274–89. doi: 10.1002/cam4.3041 (PMC7300415; doi:10.1002/cam4.3041)

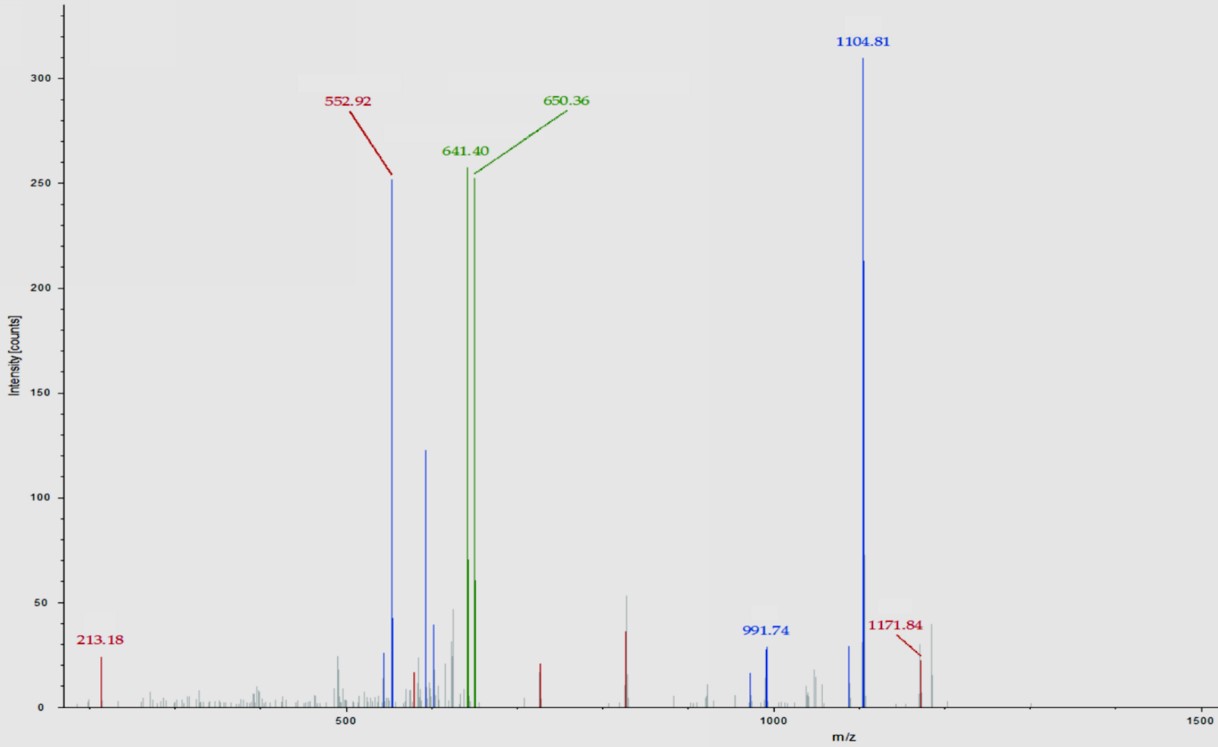

Supplement: Supplementary file 1 — Supplementary Material [file CAM4-9-4274-s001.jpg]
